# When Age Matters: How Regulatory Emotional Self-Efficacy in Managing Negative Emotions Can Mitigate the Effects of Emotional Inertia for Younger Workers

**DOI:** 10.3390/healthcare13162047

**Published:** 2025-08-19

**Authors:** Simone Tavolucci, Lorenzo Filosa, Valentina Sommovigo, Valentina Rosa, Fabio Alivernini, Roberto Baiocco, Anna Borghi, Andrea Chirico, Chiara Fini, Tommaso Palombi, Jessica Pistella, Fabio Lucidi, Guido Alessandri

**Affiliations:** 1 Department of Psychology, Sapienza University of Rome, 00185 Rome, Italy; lorenzo.filosa@uniroma1.it (L.F.); valentina.sommovigo@uniroma1.it (V.S.); valentina.rosa@uniroma1.it (V.R.); guido.alessandri@uniroma1.it (G.A.); 2 Department of Developmental and Social Psychology, Sapienza University of Rome, 00185 Rome, Italy; fabio.alivernini@uniroma1.it (F.A.); roberto.baiocco@uniroma1.it (R.B.); andrea.chirico@uniroma1.it (A.C.); tommaso.palombi@uniroma1.it (T.P.); jessica.pistella@uniroma1.it (J.P.); fabio.lucidi@uniroma1.it (F.L.); 3 Department of Dynamic and Clinical Psychology, and Health Studies, Sapienza University of Rome, 00185 Rome, Italy; anna.borghi@uniroma1.it (A.B.); chiara.fini@uniroma1.it (C.F.)

**Keywords:** regulatory emotional self-efficacy, emotional dynamics, aging at work, emotional inertia

## Abstract

**Background/Objectives**: Negative emotional inertia describes the extent to which a prior emotional state can predict the subsequent one, and it is considered a significant indicator of psychological maladjustment that has several negative consequences in the workplace. The current study tested a theoretical model in which the inertia of negative emotions is moderated by regulatory emotional self-efficacy beliefs (RESE) in managing negative affects across workers of different ages. Specifically, we hypothesized that RESE moderates the relation between negative emotions at consecutive time points, reducing their persistence, and that age would influence this relation, with older workers relying less on this resource than younger ones. **Methods**: Participants were 221 workers (57.8% females) exposed to social work stressors who reported their affectivity every evening for 31 consecutive days. We analyzed the data using dynamic structural equation models (DSEM), which enable examining within-person time series trends while estimating individual differences therein. **Results/Conclusions**: In line with our predictions, results suggest that emotional self-efficacy is a key personal resource that might be able to buffer individuals from emotional stasis, a resource primarily useful for younger workers who rely less on actual emotional regulation expertise than older adults.

## 1. Introduction

Work constitutes a central part of adult life, with full-time employees spending approximately one-third of their waking hours engaged in work-related activities [1]. Emotions, being core to human functioning, naturally interact with the work environment [2]. Defined as adaptive responses to internal and external stimuli, emotions help guide thoughts and behaviors [3,4,5,6], and workplace events frequently elicit emotional responses—whether through interactions with colleagues, leaders, or clients [7,8,9].

Understanding how emotions fluctuate and persist is essential to grasping employee well-being in organizational contexts. Building on the emotional responses described earlier, emotional dynamics—especially the persistence of emotions over time—offer a valuable lens through which to assess workplace adaptation. Three key features characterize these dynamics: baseline emotional levels, variability, and attractor strength [10]. Among them, emotional inertia—defined as the temporal persistence of emotions—has been linked to reduced adaptability and diminished self-regulation efficacy [11], underscoring its significance in modern workplaces where emotional flexibility is increasingly vital [12].

Although emotional inertia has been linked to negative psychological outcomes such as poor adjustment and burnout [13,14,15], its specific mechanisms and implications within workplace settings have received comparatively little attention. To address this gap, the present study turns to the concept of regulatory emotional self-efficacy (RESE) as a promising moderating factor. RESE—defined as individuals’ beliefs in their ability to manage negative emotional states—has been shown to conserve emotional resources and foster greater adaptability [16,17,18], making it particularly relevant for occupational contexts.

Given the centrality of emotion dynamics in the workplace and the maladaptive potential of emotional inertia, RESE emerges as a particularly relevant construct to study. As a form of self-evaluative belief, it plays a crucial role in shaping how people respond to emotional challenges, especially in work environments characterized by high social demands and emotional load. Workers with higher RESE show greater emotional flexibility and resilience, allowing them to recover more quickly from prolonged negative emotional episodes [19,20]. This is vital in buffering emotional inertia, which is often driven by ineffective regulation strategies or insensitivity to environmental shifts [21]. Thus, RESE may function as a core psychological resource in occupational contexts, helping to minimize the persistence of negative affect and enhance emotional adaptability in roles where interpersonal strain is a routine demand [14,22].

Given that emotional functioning changes with age, it is important to consider how the effectiveness of RESE may differ across the lifespan. Age may moderate the influence of RESE because emotional regulation strategies and capacities evolve with life experience. In a compensatory framework, RESE may function as a critical regulatory scaffold for younger workers who have not yet consolidated work-related mature emotional regulation strategies, while being less essential for older adults who already can count on established regulatory repertoires, thereby creating age-differentiated pathways through which perceived emotional self-efficacy influences emotional inertia.

In fact, older adults generally report fewer negative emotions and tend to use more efficient strategies, such as antecedent-focused regulation, which involves modifying situations before emotional responses are triggered [23,24]. These patterns reflect principles from Socioemotional Selectivity Theory (SST) [25,26], which suggests that aging brings a shift in motivational priorities toward emotionally meaningful experiences and relationships. SST identifies goal preferences and time perspective as key levers [27] that can be suited for fine-tuning interventions on the specific needs of workers of different ages.

In contrast, younger workers may find themselves in more demanding and uncertain career phases, marked by higher performance pressure, less job security, and the need to establish professional identity [28]. These conditions can lead to more frequent experiences of negative affect and greater difficulty in regulating emotions, particularly when regulatory strategies are not yet fully developed [29]. As such, younger adults may depend more heavily on perceived emotional regulatory resources—such as RESE—to manage their daily emotional demands. Examining these age-related dynamics is essential to fully understanding the role of RESE in work settings, as its function may vary not due to its inherent value, but because of the differing ways it is utilized across developmental stages. These insights are crucial for creating interventions and emotional support strategies tailored to the specific regulatory profiles of younger and older employees, ultimately promoting well-being and adaptability across the workforce.

To investigate these theoretical assumptions, the present study adopts an intensive longitudinal design spanning a full working month among professionals engaged in socially demanding roles. The primary goal is to explore how emotional inertia is influenced by workers’ beliefs in their own emotional regulatory abilities. Special attention is given to the role of age, as a factor potentially moderating the effectiveness of RESE in shaping emotional adaptation. By examining these dynamics within real-life occupational settings, the study aims to contribute to a deeper understanding of how individual psychological resources interact with age-related factors to support emotional flexibility and resilience at work. These insights may inform the development of age-sensitive strategies to promote well-being and adaptive functioning across diverse professional populations.

### 1.1. Emotional Inertia

Emotional inertia refers to the degree to which emotions persist from one moment to the next, demonstrating temporal persistency and continuity [21]. When inertia is high, it is often associated with a status of systemic stasis where an individual is “affectively stuck” [30] (p. 1413). Emotional inertia is usually measured using an autoregressive slope predicting a current emotional state based on the same emotion measured at a previous time point [10,31,32], with no reference to the absolute level of the experienced emotion. For example, in the case of negative emotions, emotional inertia refers to the prolonged experience of negative emotions and not the negative experience itself [33].

Emotional inertia has important implications, especially in real-life contexts where individuals need strong emotion regulation to cope with changing demands [22]. In line with this expectation, empirical studies have linked inertia with exhaustion, a core aspect of burnout syndrome [34] characterized by pervasive fatigue and drained energy [14]. Emotional inertia following exhaustion signals contributes to a cycle of resource depletion [35] that can increase the likelihood of counterproductive work behaviors (CWB) within organizations [33]. This can have physiological repercussions, for example, through its association with low heart rate variability (HRV), a biomarker of work-related stress [22]. Thus, workers with high emotional inertia may struggle particularly with workplace social challenges [36], such as interpersonal conflicts, as their negative emotional responses tend to persist even after circumstances have changed.

### 1.2. Buffering the Negative Effect of Emotional Inertia

Given these effects, it emerges that there is a necessity to study constructs that both counteract emotional inertia and can be developed within HR interventions. In this study, we focus on emotional self-efficacy [37,38], a particularly malleable cognitive construct (whose intervention sources are well established mastery experiences, vicarious experiences, verbal persuasion, and physiological states) [17], which meets those criteria necessary to effectively counteract the negative effects of emotional persistence.

Indeed, a large body of literature emphasizes the relevance of RESE regarding the management of negative emotions in fostering self-regulatory processes in various domains of psychological functioning [39]. For example, RESE has been associated with different aspects of individuals’ social functioning [40], such as fewer depressive symptoms [41], high resilience [42], and more prosocial behaviors [43]. RESE also promotes adjustment and well-being in adults, as shown by its links with positive thinking, self-esteem, optimism, and life satisfaction [42], and with emotional stability [44,45]—a personality trait strongly associated with mental health [46,47]—reflecting the ability to effectively cope with negative emotions such as stress, anxiety, discontent, irritability, and anger [48,49], and it is closely linked to burnout [45,50,51].

Specifically in the work context, recent studies have highlighted the role of RESE in promoting employee adjustment and well-being [52]. RESE has also been associated with lower emotional and cognitive resource depletion [45] and interpersonal strain [53], and with greater organizational socialization and adaptability during early career phases [20]. Moreover, RESE has demonstrated predictive value for key occupational outcomes, including reduced emotional exhaustion and stress, showing incremental validity over established constructs such as emotion regulation, effortful control, and emotional intelligence [54]. In addition, psychophysiological evidence indicates that higher RESE is associated with psychophysiological adjustment indicators at work, such as better cortisol production [55]. Collectively, these findings underscore the relevance of RESE in predicting emotional functioning and well-being across diverse professional contexts [18].

Given that individuals must invest psychological resources to enact self-regulation processes in order to modulate emotional states underlying the experience of negative emotion inertia [21], repeated experiences of daily social hassles contribute to emotional inertia through the depletion of resources for emotional regulation. This relation appears to be moderated by (a) effectively managing stress and interpersonal strain, and (b) the individual’s ability to adapt to tough circumstances [14,22,33]. Thus, analogous to regulatory processes that have been shown to play an important indirect role with respect to emotional inertia through reduced consumption of energy required for emotion regulation, such as ego-resiliency [14], RESE may play a vital role in protecting workers against the depletion of emotional and cognitive resources during emotional regulation [56].

When confronted with stressful situations, individuals who lack confidence in their capacity to regulate negative emotional states are likely to experience heightened levels of anxiety, fear, or depression [57] and, more critically, individuals who perceive themselves as unable to manage negative emotions often display these emotions in maladaptive ways [58,59]. As individuals with more robust self-efficacy demonstrate greater resilience and are capable of managing stress more effectively, persevering despite setbacks and setting increasingly challenging goals [16], RESE could buffer the impact of exhaustion and depletion on one’s emotional experience, thereby preventing the prolonged experience of negative emotions and contributing to increased well-being among workers and, consequently, the entire organization [33].

Moreover, RESE could play an important adaptive role in how more stable personality dispositions, such as traits [60], may develop and interact with the specific environment [44]. Indeed, RESE has been seen to operate at an intermediate level between general dispositions and specific behavioral tendencies [44] for the actualization of one’s own potentialities in the specific context of reference [61,62]. Thus, RESE may emerge as a key construct of individual adaptivity [18,19,20] because of its ability to evolve and shape itself based on the specific environmental demands [63]. Consequently, this adaptive mechanism could be able to buffer those processes of inflexibility to the external environment, such as the persistence of emotions [21].

To date, the buffering role of RESE on negative emotional inertia has only been explored for single emotions (e.g., anger and sadness) [64]. It has not been examined as a broader construct, encompassing varying degrees of activation (high/low arousal) [65] and not in a specific context, such as the organizational one [54].

Considering the above reasoning, we make the following hypothesis.

**Hypothesis** **1.** *RESE will moderate the relation between negative affect at time t and at time t − 1, such that higher RESE levels would result in less persistence of negative affect.*

### 1.3. The Role of Age in Affective Experiences

As noted, to effectively apply self-regulation programs in personnel management and reduce the impact of emotional inertia on employee well-being, it is essential to examine the role of age in emotional functioning. Research has demonstrated the significant impact of age on individuals’ ability to manage emotional demands [66,67,68]. This can be ascribed to several factors. First, from a personality perspective, the disposition of emotional stability appears to be the only trait to show linear growth throughout the lifespan [69], thus indicating increasing levels of this trait while aging. Since workers with lower levels of emotional stability show more anxiety, depression, nervousness [60] and more use of ineffective coping strategies such as denial, wishful thinking, and self-criticism [70,71], it could be expected that older workers may handle the emotional workload differently than younger workers by leveraging other aspects of the emotional regulation, specifically using mostly the actual emotional regulation competence and relying less on the self-efficacy domain.

Another important factor to consider in order to understand the age-related differences in the emotional domain is workers’ motivation and goals. According to SST [25,26], the key driver of these differences is time perspective: older adults perceive their time as more limited, prioritizing emotional well-being and positive experiences, focusing mostly on emotionally satisfying goals [25], and, consequently, resulting in a better daily emotional regulation compared to younger adults [72]. This improved self-regulation ability stems from several processes that occur as age increases. Indeed, older people tend (a) to prefer emotionally close and well-known social networks favoring gratifying and less frequent interactions [72,73], (b) to experience fewer negative emotions in their daily lives [23,74], and (c) to have an emotional life with greater complexity and characterized by more differentiation capabilities and at the same time with less variability and inertia [23]. All these aspects point to marked differences between younger and older adults, not only in their different abilities to self-regulate their emotional processes but also in their choice of strategies to implement through, for example, greater use of antecedent-focused emotion regulation [24,75] and less on response modulation [76] as age increases. According to this theoretical framework, some emotional regulation resources, such as self-efficacy, may be less crucial for older adults as they have developed a more comprehensive and efficient approach to emotion management that relies less on effortful control [77] and more on strategic selection and optimization of emotional resources.

Given this central role of aging in the emotional sphere, we may expect an influence of age on the relation between emotional self-efficacy and the persistence of negative emotions. Indeed, older adults may show highly efficient emotion management ability, relying on strategic selection and optimization processes, which may diminish their need for self-perception resources like emotional self-efficacy. Therefore, we make the following hypothesis:

**Hypothesis** **2.** *Age will moderate the effect of RESE on emotional inertia, with younger workers showing a stronger buffering effect of RESE compared to older workers.*

### 1.4. The Present Study

The present study aims to investigate how emotional self-regulation processes unfold in the daily lives of workers exposed to high interpersonal demands, focusing in particular on the role of RESE in moderating emotional inertia. Building on prior work linking emotional inertia to poor adjustment and burnout, and considering the buffering potential of RESE, we examine whether individuals’ beliefs in their capacity to manage negative emotions can reduce the day-to-day persistence of such negative affect.

In doing so, we also consider age as a key factor that may shape the effectiveness of RESE. Previous research suggests that while younger workers may rely more heavily on perceived regulatory resources like RESE, due to higher emotional reactivity and lower experiential maturity, older adults tend to draw upon more established regulatory strategies and motivational priorities. This study, therefore, tests whether the protective effect of RESE on emotional inertia is stronger among younger workers, for whom emotional self-efficacy may serve as a more central regulatory function.

To address our hypotheses, we employed an intensive longitudinal design, collecting daily self-reports over a full working month from a sample of professionals in socially demanding occupations. This approach allows us to assess within-person dynamics of negative affect and emotional persistence, and to test the moderating role of RESE and age in real-world occupational settings. By capturing fluctuations in emotion and their regulatory correlates in a naturalistic context, this study contributes to a finer-grained understanding of emotional adaptation at work, offering actionable insights for the development of age-sensitive emotional well-being interventions and HR strategies.

## 2. Method

### 2.1. Sample

Participants were 221 adults (57.8% females), aged about 45.81 years (SD = 12.64), with an average job tenure of 17.29 years (SD = 11.58). All worked in public-facing professions (i.e., nurses, doctors, teachers, etc.) but in all different organizations and occupational sectors: 4.3% in transportation and storage, 23.2% in commerce, 4.3% in restaurant/tourism, 2.4% in information and communication, 6.7% in financial, insurance, and real estate activities, 8.5% in professional, scientific, and technical activities, 10.4% in administrative and support services activities, 4.9% in public administration and defense, 18.9% in education, 12.2 per cent in social and healthcare assistance activities, and the remaining 4.2 per cent were employees in various other fields. No incentives were offered. Participants were recruited using multiple methods such as participants’ waiting list, advertising in the national press, posts on social networks, and the snowball technique. Individuals were first contacted, invited to take part in the study, and briefly acquainted with the general topic and purpose of the study and the possibility of resigning from participation at any moment. They were also told that participation was anonymous and that their data would be used only for research purposes. Participants who agreed to join the study received online links to complete the questionnaires.

### 2.2. Design and Procedure

To be eligible to take part in the research, participants had to be at least 18 years old, be employed for shifts of at least 5 h per day (Monday to Friday only), and be in contact with the public as part of their job role. Individuals who agreed to participate were informed of the objectives of the research project and the voluntary nature of participation. Prior to taking part in the study, all participants provided informed consent for data processing and were assured anonymity and confidentiality of their responses.

The research was divided into two phases: in the first, all participants completed a baseline survey (socio-demographic, workplace details, more stable psychological features, etc.). Then, one week later, through the daily diary design, we collected participants’ daily emotions and negative work events through an online platform where they completed the questionnaires every evening (between 6 p.m. and 10 p.m.) during 31 consecutive working days, excluding holidays and weekends. Covering an entire month allowed us to sample a reasonable number of workers’ emotional experiences without discouraging participation.

### 2.3. Measures

*Regulatory emotional self-efficacy beliefs at work at baseline* were measured with the six items of the regulatory emotional self-efficacy (RESE; ω = 0.819) [78] scale adapted for organizational contexts [54,55]. Respondents rated their perceived ability to manage negative emotions at work (e.g., “Keep calm in stressful and tense work situations” or “Overcome irritation at the wrongs suffered in your work”) on a 5-point Likert scale (1 = Not at all capable; 5 = Completely capable).

*Negative Affectivity* was measured with participants’ ratings of their levels of multiple negative emotions, reporting the degree they were experiencing each emotion using a slider scale from 0 to 100 retrieved from the Positive and Negative Affect Schedule (PANAS) [79,80]. Emotions were chosen to represent different levels of activation [81] and included sad, irritated, anxious, depressed, and stressed. Negative emotions were the starting point for calculating participants’ emotional inertia levels.

*Neuroticism personality trait at baseline* was measured using the 12 items of the Neuroticism subscale from the Big Five Inventory 2 (ω = 0.845) [82]. Examples of items are: “I am someone who can be tense “ and “Worries a lot”. The response format was a 5-point Likert scale ranging from 1 = disagree strongly to 5 = agree strongly.

*Work demands* were measured through a 5-item work demands adapted checklist (e.g., “My job required me to do things very quickly” or “Had a heavy workload”) [83]. The checklist included events that may occur frequently at work [7] and measures of job demands, such as workload, drawn from the Job Content Questionnaire (JCQ) [84]. A composite (formative) [85] index was created by summing the events (each one decoded as 0, i.e., event did not happen, or 1, i.e., event happened). Given the potential independence among the different demands, computing a reliability coefficient was not appropriate [86].

### 2.4. Statistical Analyses

We assessed our hypotheses using multivariate Dynamic Structural Equation Modeling (DSEM) [87]. DSEM integrates the analysis of Time Series Analysis, Multilevel Modelling, and Structural Equation Modelling [88]. This approach allows for the distinction between associations at the between-person level (reflecting stable interindividual differences) and the within-person level [89], thereby avoiding the confusion between trait and state variance [90] while modeling within-individual relations across single time series data.

Furthermore, DSEM incorporates latent mean centering, which mitigates biases linked to observed person-mean centering [91]. Moreover, it enables the modeling for each individual of random residual variances that fluctuate within individuals, enhancing the approximation of single differences in predictability. In this study, we applied DSEM to investigate a moderated model in which emotional self-efficacy predicts emotional inertia, with age-related differences moderating this relation.

**Figure 1:**
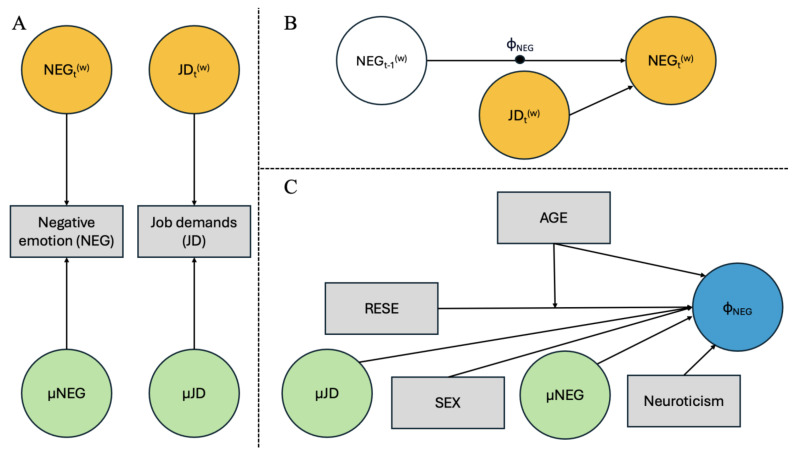
Illustrative dynamic structural equation modeling for the second model tested. (**A**) = Latent variance decomposition in within and between-individual components; (**B**) = within-individual dynamic model; (**C**) = between-individual differences model.

To achieve this, we implemented a two-level autoregressive lag-1 model (AR [1] model) [92]. The model estimated the within-person level (or Level 1) autoregressive path linking negative emotions on a day (NEG_t − 1) to next-day negative affectivity (NEG_t). The autoregressive path was treated as a latent variable (i.e., random slope) with a mean and variance estimated at the between-person level (or level 2). Consequently, the mean represents the fixed effect capturing the average effect across persons, and the random effect represents the variance of person-specific deviation around this. At this level, the mean of negative emotions, representing the average level of the construct, as well as its variance, representing between-person deviations from average construct levels, were estimated. The interaction of RESE and age was added at Level-2 as a predictor of the random coefficient of emotional inertia, capturing the average strength of prediction of inertia by RESE on age-related basis. Control variables were added at Level-2 as predictors of inertia of negative emotions (job demands, mean level of negative emotion, sex, neuroticism) to obtain more refined estimates (see Figure 1).

We analyzed the data in MPLUS 8.3 [93] using Bayesian estimation with the default uninformative prior distributions with 3000 iterations of the Markov chain Monte Carlo algorithm and the thinning factor set to 2. We used the 95% credible intervals to analyze the effect’s statistical significance. When the credible intervals did not include zero, they were considered significant estimates [94,95]. In order to take into account the unequal interval between measurement occurrences (no questionnaires were sent during weekends and holidays) and the missing values, we set the TINTERVAL option as 1 [92]. Uninformative priors were employed to (a) allow the data to primarily drive posterior estimates, minimizing the influence of researchers’ assumptions [96], given the lack of strong theoretical foundations to inform prior distributions [92]; and (b) facilitate replicability and comparability across studies [96].

We calculated within-individual standardized parameters for emotional inertia [97]. This parameter represents the average of standardized within-individual estimates. The effect size was interpreted as small, medium, and large for values of 0.10, 0.30, and 0.50, respectively. Because it represents between-person differences in intra-individual estimates, effect size could not be calculated for the random effect.

The reliabilities of the daily measures of job demands and negative emotions were estimated by using the multilevel indices ICC (1) and ICC (2). The ICC (1) is a measure of score reliability over time, whereas the ICC (2) indicates the proportion of the total variance that is accounted for by stable, trait-like between-person differences.

## 3. Results

### 3.1. Descriptive Statistics

Table 1 provides descriptive statistics for all constructs across days of assessment and participants, as well as correlations among constructs at the within—and between-person levels. All the ICC values showed high values—especially the ICC2 with values above 0.90 for the two variables—suggesting a high degree of between-person variance and thus large differences among time series of different persons.

Between-individual correlations among RESE and negative emotions (the latter obtained by averaging the participants’ scores across the 31 days) showed a negative and significant correlation (r = −0.315, *p* < 0.001). RESE showed the only other significance with Neuroticism (r= −0.564, *p* < 0.001). The same two variables, NEG and NEUR, showed negative and significant correlations with age (−0.247, *p* < 0.005; −0.195, *p* < 0.05, respectively). Finally, regarding the within-individual correlation between job demands and negative affectivity, the association was significant and positive with a value of 0.128 (*p* < 0.001), showing an intraindividual relation for the two variables taken on the same evening.

### 3.2. Moderation Model

The model converged efficiently according to the above criteria. The model achieved excellent convergence, with a final Potential Scale Reduction (PSR) value of 1.002 [98,99]. The effective number of parameters was 7780.825, reflecting the individualized parameter estimation characteristic of DSEM approaches, while the deviance was 21,272.012 [96].

In this model, we calculated the inertia of emotions at within level (Φ_NEG_), through an autoregressive path (NEG_t − 1 to NEG_t) with mean and variance estimated at level 2 (see Table 2).

At the within level, Φ_NEG_ emerged as significant with a value of 0.259 (*p* < 0.001, CI 95% = 0.220, 0.294) indicating how much, on average, workers’ deviations from their mean at time t − 1 predict deviations at time t, i.e., that the prior emotional level explains 25.9% of the emotional variance at any given time. The residual within variance of NEG was significant, with a value of 0.882 (*p* < 0.001, CI 95% = 0.852, 0.904). At this level, as mentioned above, an additional nonlagged regression was added examining the influence of negative events and job demands on negative emotions. This relation yielded a positive and significant value of 0.122 (*p* < 0.05, CI 95% 0.096, 0.146). The within-level R-square averaged across clusters was 0.118 (*p* < 0.001, CI 95% = 0.096, 0.148).

At the between-individual level, we could see a small/medium effect of RESE on Φ_NEG_ (r = −0.266, *p* < 0.05, CI 95% = −0.475, −0.001). Consistent with our H2, the interaction term of RESExAGE was also significant, showing a large effect (r = 0.509, *p* < 0.005, CI 95% = 0.135, 0.752), attesting that the protective effect of RESE on emotional inertia varies according to the age of the participants. In contrast, age alone did not seem to directly play a determining role (r = −0.158, *p* > 0.05, CI 95% = −0.343, 0.088). No covariates evidenced a significant value. Finally, negative emotion inertia resulted in having both significant intercept (r = 0.712, *p* < 0.05, CI 95% = 0.092, 1.507) and residual variance (0.533, *p* < 0.001, CI 95% = 0.276, 0.829). The between-level R-square was 0.467 (*p* < 0.001, CI 95% = 0.170, 0.724), which indicates that the model explained about 47% of the between variance of this dynamic index.

## 4. Discussion

**Figure 2:**
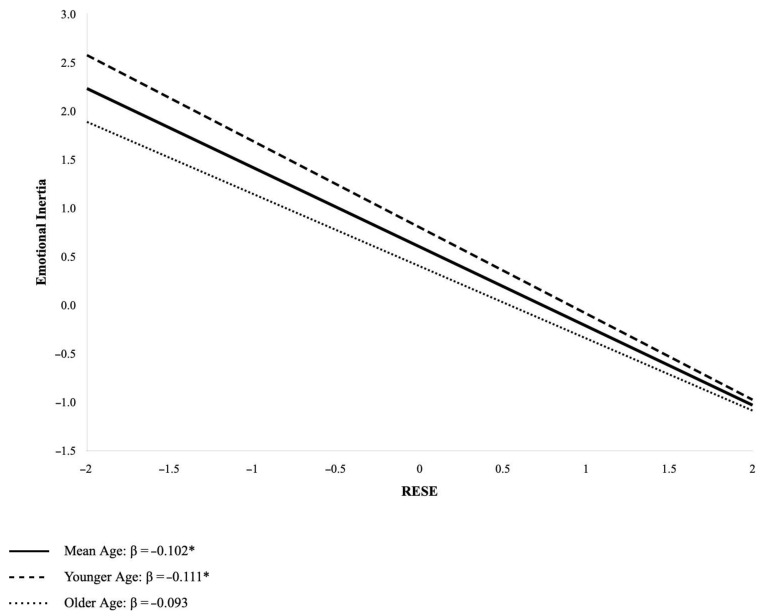
Effects of RESE on emotional inertia at different age levels. Note. * *p* < 0.05.

The results confirmed our hypotheses. In our model, RESE is found to play a protective role regarding the persistence of negative emotions in the workplace, confirming our Hypothesis 1. Even more central is the relation with age. As shown in Figure 2, this buffering function of emotional self-efficacy on the persistence of negative emotions is moderated by age. These results support our Hypothesis 2. Younger workers make greater use of self-efficacy as a regulatory resource to buffer the inertia of negative affectivity. This may occur for several reasons. Younger workers face a different career stage than older workers, leading them to allocate their resources differently. While younger workers must focus on building their careers and thus, in accordance with the Selection, Optimization, and Compensation theory (SOC theory) [100], be primarily devoted to growth and maintenance goals while defining and developing their professional competencies, older workers may exclusively focus on maintenance and regulating loss activities [101,102,103]. This developmental stage requires a considerable investment of resources, making younger workers more susceptible to resource depletion due to daily hassles, and in this context, self-efficacy may play a more salient role.

Another important age-related difference involves individual characteristics and specifically the different ability to emotionally self-regulate between younger and older people, with the latter showing greater self-regulatory capacities [68,72,104,105], but also a different use of regulatory strategies. Several studies converge in pointing out that older people may enact a minor use of suppression strategies [76,106], a strategy less effective and often correlated with maladjustment indices [107,108] that involves the effort to reduce or eliminate an emotional response after it has been triggered. On the contrary, older people have been seen to use more reappraisal processes [109,110], a strategy considered more adaptive and focused on cognitively reframing a situation in order to reduce its emotional intensity. This is consistent with SOC-ER theory [75], which points out that even within the sphere of emotional regulation there is greater benefit for the individual in using those strategies that maximize available resources. In this sense, the efficiency of emotion regulation strategies is contingent upon an individual’s available regulatory resources across domains such as cognitive control associated with aging. According to the SOC-ER theory, enhanced cognitive control may amplify the benefits of cognitively demanding strategies such as reappraisal. Consequently, the use of more functional regulatory strategies together with a more pronounced ability to control one’s own temperamental impulses and a higher level of emotional stability [69] may have a strong impact on another cognitive process such as self-efficacy, which may consequently assume a more marginal role as older people already implement more effective and adjusted regulatory strategies.

For the above reasons, future research should focus on emotional domain aspects, such as the use of adaptive and maladaptive emotion regulation strategies, to better capture the specific components of emotional inertia that may result from their implementation. Furthermore, it will be important to investigate the impact of more stable individual characteristics, such as affective temperament, defined as stable and genetically determined modalities through which individuals experience emotions and affect [111], and effortful control, a construct referring to cognitive and behavioral temperamental self-regulation, involving the ability to inhibit a dominant response and activate a subdominant one, thereby supporting the management of impulses, emotions, and attention [112].

### Limitations

This study presents several strengths, including the use of a longitudinal design covering a considerable number of working days (31 consecutive working days) and a multilevel statistical approach capable of modeling and distinguishing within-individual variance, in order to calculate the dynamic index of emotional inertia, from between-individual variance. Furthermore, the inclusion of a comprehensive set of control variables enhanced the accuracy of our analyses.

However, the study is not without limitations, and these offer insights for future research.

Firstly, the dynamic data on negative emotions were collected only once daily. While this choice allowed us a month-long assessment period and the recruitment of a large sample, it precluded us from being able to capture finer fluctuations in daily emotional dynamics [10].

A second limitation concerns the measurement of RESE. While we employed a between-individual measure of this scale in order to study more “trait” and stable aspects of this construct [64], a dynamic measurement of this scale would have enabled the observation of within-individual variations in emotional self-efficacy, providing further insight into the functioning of these malleable cognitive structures [113,114,115]. Indeed, self-efficacy may benefit from regulatory mastery experiences, which serve as a crucial source for the development of these beliefs [17]. This could create differentiated paths based not only on age-related differences but also on successful daily self-regulative experiences.

A third methodological issue regards the use of TINTERVAL = 1 function. In our dataset, we included Friday-to-Monday transitions (accounting for approximately 20% of the autoregressive process) that may reflect qualitatively different dynamics compared to the other weekday-to-weekday paths. Future studies could benefit from approaches that explicitly model both intra-weekday processes and weekend recovery transitions to achieve a more comprehensive understanding of work-related emotional rhythm patterns.

Another limitation regards the exclusive use of self-report measures, which may lead to results biased by the common method variance [116], although several methodological countermeasures were implemented in our research design to reduce its effect [117,118]. While self-report is relevant for constructs such as self-efficacy (which relies on individual beliefs) [63] and negative affect [119], measures such as job demands might benefit from alternative methodologies, such as hetero evaluations (for example, by supervisors or team members).

Finally, a further limitation involves the nature of the participants’ work activities. Although all participants were selected only if they were engaged in public-facing roles, potentially exposing them to similar social stressors, a larger sample would have facilitated more refined distinctions regarding the types of interactions they encountered within their work environment. This would allow for a clearer differentiation between demands associated with, for example, healthcare (as doctors in contact with patients) and educational settings (as teachers in contact with students).

## 5. Conclusions

In this study, we aimed to analyze the role of self-efficacy in managing negative emotions (RESE) in moderating the persistence of negative affectivity in a sample of workers in public-facing jobs for 31 consecutive days. We hypothesized that this role of self-efficacy may depend on the workers’ age, given that at different ages people not only face different work phases that underlie a different level of job demands and goals, but also cope with emotional regulation more (or less) effectively also based on the different use of regulation strategies, with older people showing greater expertise in regulation skills and greater use of reappraisal (than suppression), which is considered a more effective strategy of regulation, with positive impacts on individual adjustment. Our hypotheses were confirmed. Emotional self-efficacy appears to play an important role in protecting from the inertia of negative emotions in our sample of workers, and this role seems to rely on the age of people, with older workers showing a nonsignificant relationship between perceived self-regulatory skills and persistence of negative emotions. According to our hypothesis, these cognitive belief structures related to one’s own regulatory abilities are a more salient and consistent aspect of younger people, with older workers relying instead on actual regulatory competencies. These results should lead to differentiated, age-specific interventions in the workplace. While for younger workers, organizations should implement emotional self-efficacy development initiatives that combine mastery experiences through simulations, vicarious modeling via mentoring, and social persuasion through constructive feedback training [17]. Older workers may benefit more from interventions aimed at reducing the use of maladaptive regulation strategies and promoting the implementation of more effective and adaptive ones (e.g., mindful awareness at work or positive reappraisal therapy) [105,120,121].

## Figures and Tables

**Table 1:** Descriptive statistics.

	(1)	(2)	(3)	(4)	(5)	(6)
1. SEX	1	—	—	—	—	—
2. AGE	0.087	1	—	—	—	—
3. RESE	−0.073	0.076	1	—	—	—
4. NEUR	0.132	−0.195 *	−0.564 ***	1	—	—
5. JD	−0.038	−0.100	0.049	0.006	1	0.128 ***
6. NEG	0.098	−0.247 **	−0.315 ***	0.491 ***	0.194 *	1
Mean	—	45.809	3.318	2.884	0.458	22.894
SD	—	12.605	0.614	0.638	0.720	19.753
ICC1	—	—	—	—	0.368 ***	0.682 ***
ICC2	—	—	—	—	0.945 ***	0.984 ***

Note. *** *p* < 0.001; ** *p* < 0.005; * *p* < 0.05. Within (i.e., state) individual variables are above the main diagonal. Between (i.e., trait) individuals’ correlations are below the main diagonal. Means and standard deviations for job demands refer to variations in averaged within-person variables between participants. ICC = Intraclass coefficients, type1 (ICC1) and type2 (ICC2); RESE = Regulatory emotional self-efficacy for negative emotions; NEUR = Neuroticism; JD = Job demands; NEG = Negative emotions.

**Table 2:** Model within/between estimates.

Parameters			Standardized Estimates	95% CI Lower	95% CI Upper
Within-level					
NEG_t−1_	→	NEG_t_	0.259	0.220	0.294
JD	→	NEGt	0.122	0.096	0.146
Between-level					
RESE	→	φ_NEG_	−0.266	−0.475	−0.001
RESExAGE	→	φ_NEG_	0.509	0.127	0.731
AGE	→	φ_NEG_	−0.158	−0.343	0.088
SEX	→	φ_NEG_	0.003	−0.209	0.219
NEUR	→	φ_NEG_	−0.141	−0.361	0.117
JD	→	φ_NEG_	−0.118	−0.315	0.067
μ_NEG_	→	φ_NEG_	0.054	−0.156	0.263

Note. RESE = Regulatory emotional self-efficacy for negative emotions; NEUR = Neuroticism; JD = Job demands; NEG = Negative emotions; Φ_NEG_ = Negative emotional inertia; μ_NEG_ = Negative emotion mean.

## Data Availability

The raw data supporting the conclusions of this article will be made available by the authors on request.
